# Severe pain and hypertension following intravesical instillation of formalin necessitating epidural analgesia

**DOI:** 10.4103/0019-5049.72660

**Published:** 2010

**Authors:** Anjolie Chhabra, Krithika Krishnan

**Affiliations:** Department of Anaesthesiology and Intensive Care, All India Institute of Medical Sciences (AIIMS), New Delhi, India

Sir,

A 75-year-old, American Society of Anesthesiology Grade II male patient, a known case of carcinoma of the urinary bladder, who had previously undergone trans-urethral resection of the bladder tumour, received intravesical BCG and six cycles of radiotherapy, was referred to our hospital with persistent hematuria for seven months. A diagnosis of post-radiotherapy haemorrhagic cystitis was made. The patient twice underwent cystoscopy with clot evacuation for the same under subarachnoid block. However, the hematuria persisted, necessitating repeated transfusions of packed Red Blood Cells. As a last-ditch effort to stop the diffuse bleeding, an old technique of intravesical instillation and irrigation with formalin was planned.[1]

The patient was a diabetic on insulin. He had no other comorbid disease. His preoperative haemoglobin was 7.4 g% and coagulation profile was within normal limits.

In the view of the short duration of the procedure, general anaesthesia was planned. Following intravenous fentanyl (60*μ*g) and propofol (100 mg), a Pro-seal laryngeal mask airway was inserted. Anaesthesia was maintained with O_2_, N_2_O, isoflurane. The patient was placed in lithotomy position and 4% formalin was instilled intravesically. Formalin was kept *in situ* for 20 min, after which the bladder was evacuated. There was an immediate hypertensive response following formalin instillation, the blood pressure rose from a baseline value of 110/80 mmHg to 190-180/120-100 mmHg with a pulse rate of 86 beats/min. The hypertensive response persisted despite repeating fentanyl (90 *μ*g). After the procedure, on awakening, the patient complained of severe, unbearable pain in the suprapubic region despite repeated doses of IV fentanyl and morphine. His blood pressure continued to remain high. To alleviate his pain an epidural catheter was inserted in the L2-3 space and 10 ml of 0.125% bupivacaine administered. After 20 min the pain subsided and his vitals returned to normal.

Formalin has a dessicating effect when applied to living tissue; it hydrolyzes proteins and coagulates superficial tissue. This effect controls the haemorrhage from telangiectatic capillaries in the mucosal and submucosal layers.[1] Sloughing of the urothelium, local oedema and inflammation cause severe pain. Regeneration can take up to threeweeks. Suprapubic pain, dysuria, a reduction in bladder capacity, urgency and incontinence are known complications.[1] The severity of the pain requires that, where possible, the procedure should be carried out under regional anaesthesia. Not many anaesthesiologists may be aware of the severe pain engendered by the intravesical instillation of formalin and we wanted to share our experience. We came across only one other report where suprapubic pain following formalin instillation was managed with intravesical lidocaine.[2] However, the duration of pain relief with such a technique would be limited. An epidural catheter offers the advantage of repeated dosing which can be beneficial, as our patient needed epidural morphine for two days.

## References

[1] Dewan AK, Mohan GM, Ravi R (1993). Intravesical formalin for hemorrhagic cystitis following irradiation of cancer of the cervix. Int J Gynaecol Obstet.

[2] Mishra S, Singhal AK, Kannan TR, Bhatnagar S (2004). Intravesical lidocaine for formalin-induced suprapubic pain. Am J Hosp Palliat Care.

